# Adult pancreatic cavernous hemangioma: case presentation of a benign tumor with a complex composition

**DOI:** 10.1186/s12876-019-1119-5

**Published:** 2019-11-27

**Authors:** Tao Lianyuan, Wang Yafeng, Yu Haibo, Dong Yadong, Ma Jiahao, Lu Yuanxiang, Li Deyu

**Affiliations:** Department of Hepatobiliary Surgery, Henan Provincial People’s Hospital, People’s Hospital of Zhengzhou University, School of Clinical Medicine, Henan University, No. 7 Weiwu Road, Zhengzhou, 450003 China

**Keywords:** Cavernous hemangioma, Pancreas, Adult, Immunohistochemical

## Abstract

**Background:**

Pancreatic cavernous hemangioma is an extremely rare benign tumor that is difficult to diagnose on an imaging examination, and its histopathological examination has rarely been reported.

**Case presentation:**

Herein, we present the case of a 63-year-old man who was admitted to the hospital due to left upper abdominal pain and defecation unformed for more than 2 years. None of the positive results obtained from the physical examination could explain his symptoms. The imaging examination indicated a multilocular cyst with septa in the head of the pancreas. The patient underwent a pancreaticoduodenectomy, and the pathologic diagnosis was pancreatic cavernous hemangioma. The histopathological examination showed that the lesion was positive for benign vascular markers, such as CD31, CD34 and F8, and negative for lymphocyte markers, such as D2–40. Moreover, it was also positive for ERG and cytokeratin markers, CAM5.2 and AE1/AE3, indicating the complexity of its components, and Ki-67 negativity revealed its benign nature.

**Conclusions:**

Pancreatic cavernous hemangioma has a complex composition that may be reflected not only in the imaging examination but also in the immunohistochemical detection, and it may achieve a good outcome by surgical excision.

## Background

As a congenital malformation of the vascular system, cavernous hemangioma is an uncommon type of primary cystic neoplasm. The occurrence of pancreatic hemangiomas in adults is extremely rarely reported [1–21], especially that of cavernous hemangioma [9, 15, 21]. Adult pancreatic hemangiomas often manifest as large cystic lesions in middle-aged females, and in many cases, the patients exhibit abdominal pain but no evidence of malignancy [11, 13, 16, 19]. The major symptoms of cavernous hemangioma are abdominal pain and distension associated with the enlarged tumor [1, 7, 11].

The diagnosis of cavernous hemangioma remains controversial [1, 7, 11]. The expression of surface molecular markers is still unclear. In the present study, we analyzed the clinical features and immunohistochemical data of a rare case of an adult patient with pancreatic cavernous hemangioma without recurrence over 2 years following curative surgery.

### Case report

A 63-year-old man was admitted to the hospital due to left upper abdominal pain and defecation unformed for more than 2 years. There was no history of acute pancreatitis or abdominal trauma. The patient had no prior surgeries. He was on no medications. The family history was noncontributory. The physical examination was normal.

**Fig. 1:**
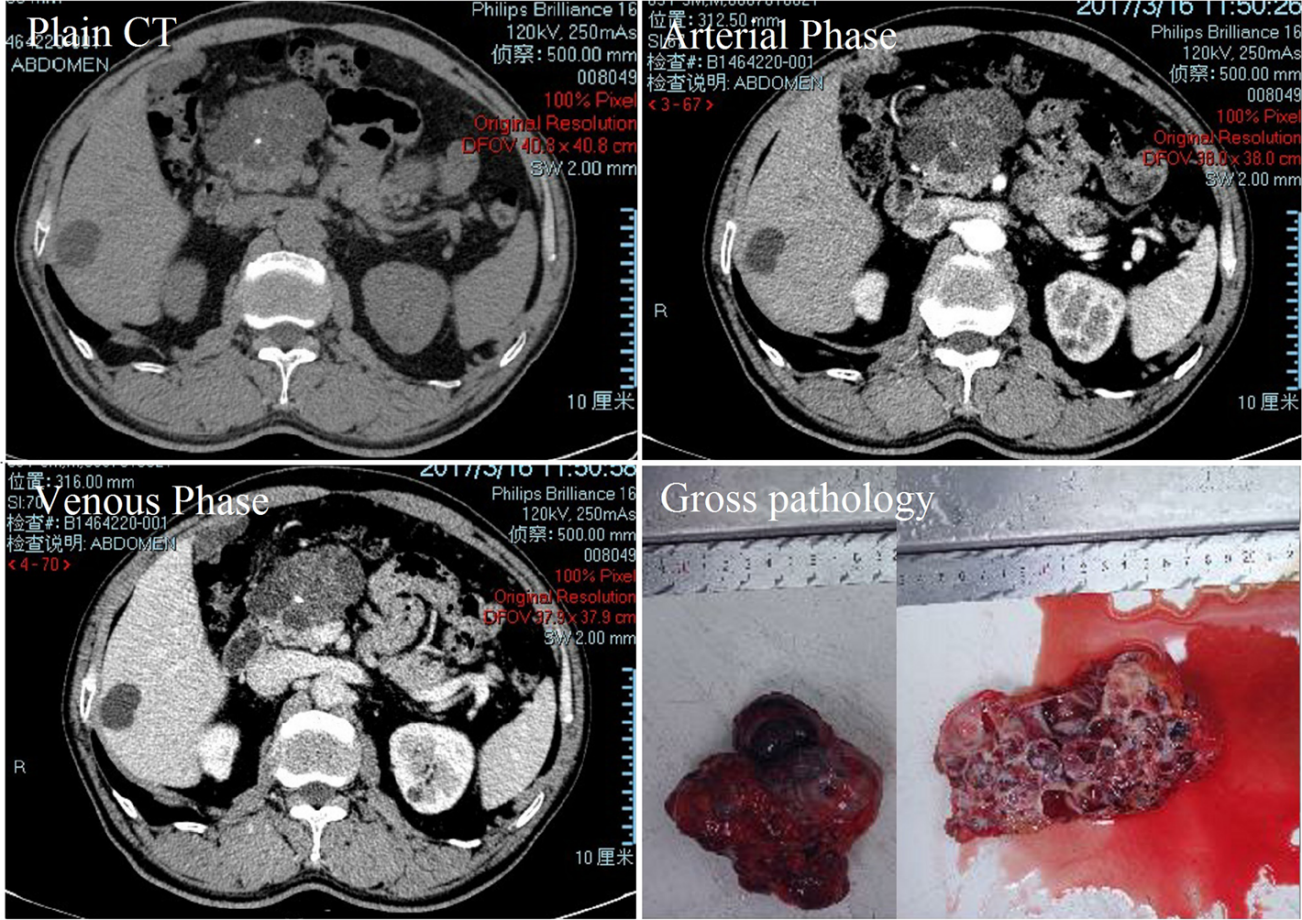
The tumor was a multilocular cyst with septa and fluid-fluid levels. Enhanced scan showing “fast in, slow out”. CT enhancement (arterial phase and venous phase): The CT value was slightly higher than that of plain CT after contrast agent injection at the base of the tumor. Gross pathology showed a cystic lesion with thick septa

Endoscopic ultrasonography (EUS) showed a mixed echo with a range of 105 × 82 mm (mainly a cystic echo with visible separation and a visible range 59 × 47 mm heterogeneous hyper echo) within an unclear contour of the head of the pancreas. CDFI indicated a high echo and visible blood flow within the separation. CT revealed a well-defined cystic mass in the head of the pancreas, with the formation of internal calcifications (Fig. 1).

Laboratory tests showed that the complete blood count, complete metabolic panel, serum amylase and lipase, coagulation panel, fasting lipid profile, and serum CA 19–9 and CEA levels were normal. The detailed results were as follows: leukocyte count 6.89 × 10^9^/L, neutrophilic granulocyte count 4.31 × 10^9^/L, neutrophil percentage 62.5%, lymphocyte count 2.03 × 10^9^/L, lymphocyte percentage 29.5%, RBC 4.6 × 10^12^/L, hemoglobin 146 g/L, platelet 163 × 10^9^/L, alanine aminotransferase 15 μL, aspartate aminotransferase, albumin 37.5 g/L, total bilirubin 9.5 mol/L, direct bilirubin 2.7 μmol/L, alkaline phosphatase 68 U/L, glutamyl transpeptidase 15 U/L, HBsAg(−), HBeAg(−), HBeAb(−), HBcAb(−), HCV-Ab (−), and CEA 14.01 ng/mL. AFP and CA199 showed no abnormalities.

A pancreaticoduodenectomy was performed, and a pink-brown, multiloculated and approximately 10 cm × 5 cm × 5 cm cystic solid mass with a clear boundary and a firm texture was found at the head of the pancreas (Fig. 1). No evidence of invasion of the portal vein or the superior mesenteric vein or other vessels was found. No bleeding occurred during or after the operation, and the patient had a smooth postoperative recovery.

**Fig. 2:**
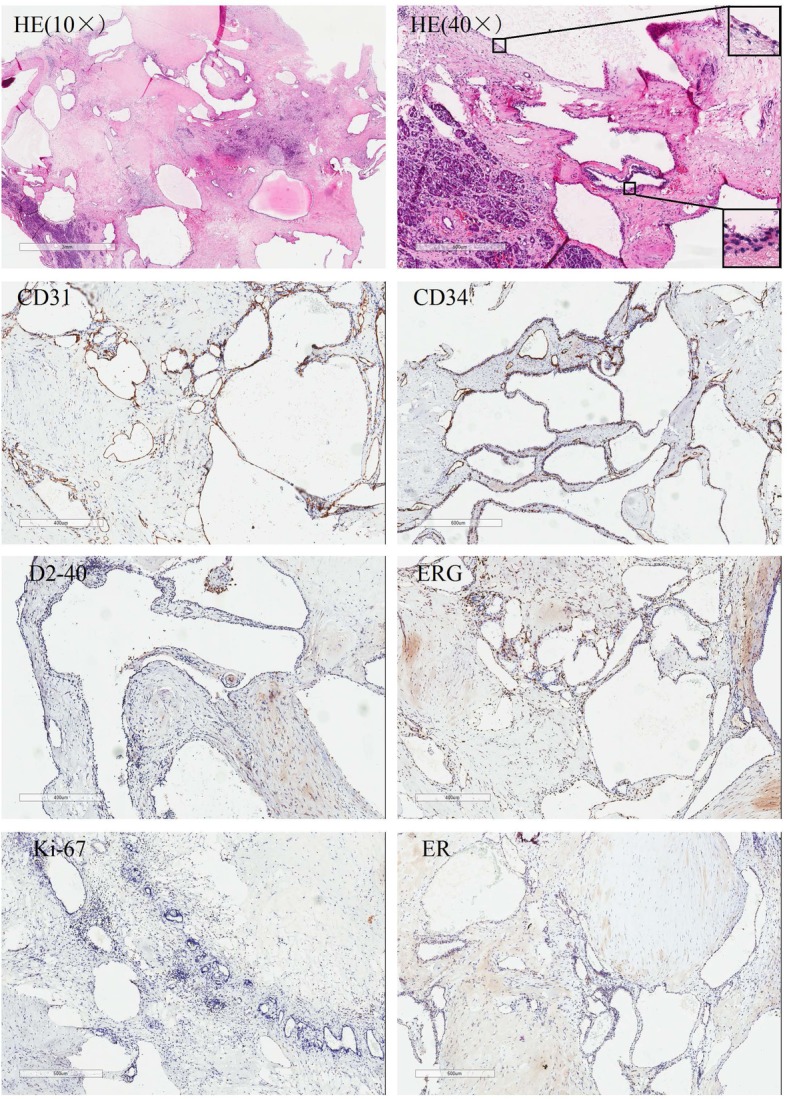
H&E staining of the tumor showing a cyst extending into the interlobular septa of the pancreatic parenchyma (HE 10×). Some of the vessels in the tumor were well-identifiable arteries with thick walls characterized by profusely proliferating multilayer endothelial cells; other vessels had thin, membranous walls with a single layer of flattened cells (HE 40×). Immunohistochemical staining indicated positivity for CD31 and CD34, focal positivity for ERG, and negativity for D2–40, ER and Ki-67 (40×)

**Fig. 3:**
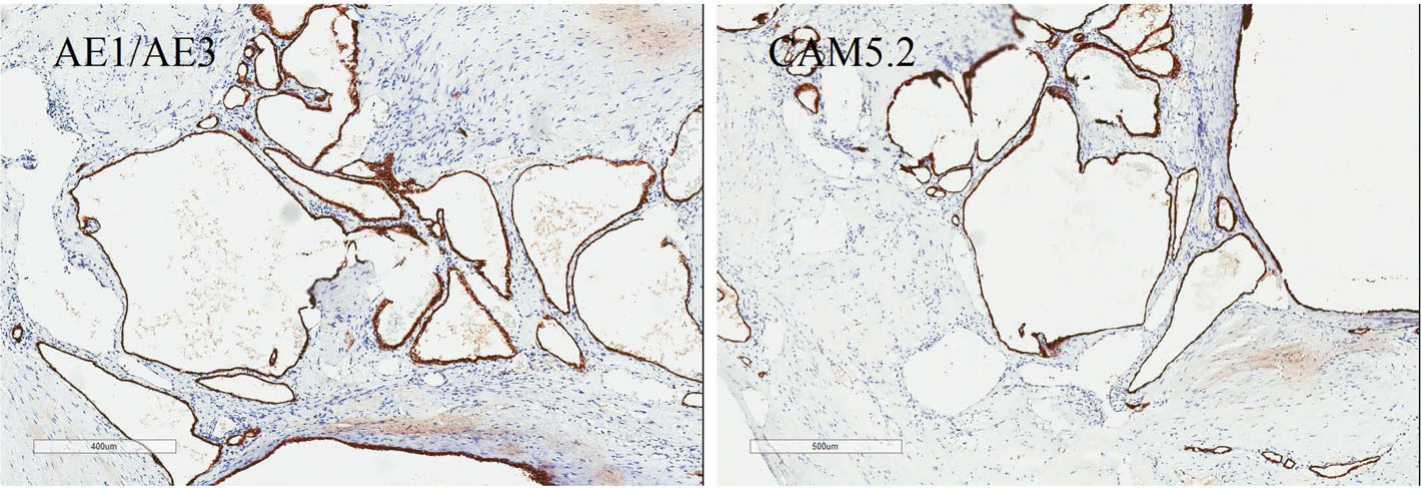
Immunohistochemical staining showing a cavernous, ectatic endothelial neoplasm positive for CAM5.2 and AE1/AE3(40×)

The microscopic examination indicated a cystic structure extending into the interlobular septa of the pancreatic parenchyma that consisted of dilated vascular structures lined by endothelial cells. Some of the vessels with a membranous wall were filled with serous fluid rather than blood, indicating that those vessels do not participate in the circulation (HE 10×). Some of the vessels in the tumor were well-identifiable arteries with thick walls characterized by profusely proliferating multilayer endothelial cells; other vessels had thin, membranous walls with a single layer of flattened cells (HE 40×), with uniform nuclei and no atypical nuclei (Fig. 2). Immunohistochemical (IHC) staining showed that the lining was positive for CD31, CD34 and F8, focally positive for ERG, and negative for D2–40, ER and Ki-67, supporting the diagnosis of hemangioma (Fig. 2). However, the cytokeratin markers, CAM5.2 and AE1/AE3, were also positive (Fig. 3). All 12 lymph nodes had a normal histology. Postoperatively, the patient tolerated an oral intake of liquids on the third day, was discharged on the 7th postoperative day and remained symptom free 2 years after surgery.

## Discussion and conclusions

Vascular tumors include lymphangiomas, hemangiomas, lymphangiomas, angiotheliomas, angioblastomas, and angiosarcomas, are of which all cystic lesions. As a benign neoplastic lesion with slow growth and rarely malignant changes, these lesions are commonly found in the skin, eyes, liver, brain, spleen and other organs, while cavernous hemangioma occurring in the pancreas is extremely rare [22]. Pancreatic cavernous hemangioma is more common in women than in men (the male to female ratio is 1:3), the exact cause of the disease is unknown, and it is mostly recognized as a congenital disease [11, 15]. The age of the patients at diagnosis ranges from 18 to 79 years, and it occurs more frequently in childhood than adulthood [1–21]. It progresses slowly to the stage of degeneration after hyperplasia in infancy and gradually disappears after many years, leaving traces of fiber and fat in adulthood [11, 15, 23]. Adult pancreatic hemangioma is very rare, and its pathological nature is completely different from that of childhood pancreatic hemangioma [11, 15, 23].

Cavernous hemangioma of the pancreas shows no typical clinical symptoms, and the majority of patients experience pain and discomfort in the middle and upper abdomen [11, 15]. Nausea, abdominal distention, fever, episodic dizziness and palpitations may be associated with pain. Some patients come to hospital because of low back pain, a palpable abdominal mass or gastrointestinal bleeding [2]. Occasionally, it may be diagnosed from incidental findings on an imaging examination during a physical examination. Neither episodes of pancreatitis nor a family history of pancreatic diseases has been reported before. It may rarely be observed in the setting of von Hippel-Lindau disease, especially in young patients [24].

The imaging manifestations of pancreatic cavernous hemangioma are diverse. Abdominal X-ray and cholangiography are of little significance in the diagnosis of pancreatic cavernous hemangioma, while digital subtraction angiography (DSA) is invasive and rarely used [15]. Pancreatic cavernous hemangioma is usually characterized by ultrasonography as well demarcated and appearing overall hyper- or isoechogenic to the rest of the pancreas with mixed echogenicity or an irregular low echo and usually no blood flow signal or a low-velocity blood flow signal. Contrast-enhanced ultrasound has revealed that cavernous hemangiomas are typically rich in blood supply, but a large portion of pancreatic cavernous hemangiomas do not show obvious enhancement, so the nature of the lesion cannot be determined.

CT and MRI are the main imaging examinations used to diagnose cavernous hemangioma of the pancreas [9, 15, 21]. Typically, hemangiomas can be significantly enhanced in the arterial phase of the CT scan, but pancreatic cavernous hemangioma is a cystic tumor that usually contains neurovascular components and is accompanied by an arteriovenous shunt, and the flow rate is slow when the blood flow passes through, so it may lead to no enhancement in the arterial phase. At the same time, the proportion of cystic and solid components in the tumor is different, which may lead to different degrees of enhancement in the arterial phase [25]. The CT manifestations of the present case were an atrial cystic mass, moderately enhanced in the base of the mass, but the mass itself did not show significant enhancement. Many studies [2, 9, 15, 25] have shown that pancreatic cavernous hemangioma does not necessarily show significant enhancement in the arterial phase of the CT scan, so it is believed that the diagnosis of this disease cannot be ruled out without obvious enhancement in the arterial phase. Moreover, in patients with pancreatic cavernous hemangioma, only a very small number of patients can present characteristic changes, and the vast majority of imaging diagnoses are difficult. Therefore, it may be easy to confuse pancreatic cavernous hemangioma with other pancreatic lesions (such as pseudocysts, serous cystadenomas, mucinous cystadenomas and intraductal papillary mucinous neoplasms, IPMNs) [7]. There was no enhancement of pancreatic pseudocysts on the CT examination. The CT findings of serous cystadenoma and mucinous cystadenoma are similar to those of pancreatic cavernous hemangioma. The margin of a mucinous cystadenoma is smooth, with or without septa, and is usually surrounded by eggshell-like calcifications. IPMN is a pleomorphic cystic mass, and the key of its diagnosis is communication with the main pancreatic duct. In addition, cavernous hemangioma of the pancreas should be differentiated from other diseases of the pancreas that are rich in blood supply, such as neuroendocrine tumors, metastatic renal cell carcinomas, an intrapancreatic accessory spleen and arteriovenous malformation [23]. A comprehensive review of the relevant literature revealed the following points to aid in the diagnosis [9, 15, 21], 1) no skin or scleral yellow staining; (2) tumor markers were mostly negative; (3) CT or MRI examination revealed mostly a circular mass with a clear boundary and the coexistence of cystic and solid components, no main pancreatic duct dilation, and enhanced scan lesions can be slightly enhanced; (4) ultrasound examination showed a high-echo mass, no blood flow signal or a low-speed blood flow signal; and (5) no lymphatic or distant metastasis. In short, cavernous hemangiomas typically have a characteristic manifestation in CT or MRI of an enhancement feature described as “fast in and slow out”, which indicates that edge nodular and patchy enhancement appear at the arterial stage, and the enhancement range spreads to the center at the portal vein stage, which is followed by delayed scanning maintained at an equal or slightly high density.

At present, the gold standard for the diagnosis of pancreatic cavernous hemangioma is still a pathological diagnosis. Microscopically, cavernous hemangioma is mainly composed of a dilated abnormal sinus, lined with monolayer vascular endothelial cells, and the fibrous tissue in the sinus is not completely spaced to form a cavernous structure [11, 15]. Depending on the size of the vascular spaces, they can be capillary or cavernous. Positivity for CD31 and CD34 indicates hemangioma, lymphangioma and other benign vascular tumors, and negativity for D2–40 and a lack of lymphocytes help to exclude lymphangioma [9, 15, 21]. Focal positivity for ERG and negativity for Ki-67 indicate the complexity of the components and the benign nature of the tumor. Our study denies the association between cavernous hemangiomas and increased levels of estrogen or negativity for ER. However, as markers of cytokeratin, which is supposed to be negative in hemangiomas [1], CAM5.2 and AE1/AE3 were positive in the present case, which may also suggest a tendency toward epithelialization and indicate the complexity of tumor components. A precise histopathological diagnosis of hemangioma is of great significance, as other vascular tumors may require more radical surgical margins at resection, adjunctive therapy and closer postresection follow-up. For example, the recurrence rate of hemolymphangioma is as high as 10 to 27% after complete resection [26].

Surgical excision is still the best treatment because almost all resected pancreas hemangioma cases have good outcomes, with the resolution of symptoms and no tumor recurrence [9, 15, 21]. However, because a preoperative qualitative diagnosis is very difficult, the choice of treatment is also very controversial. By fully understanding the imaging characteristics of pancreatic cavernous hemangioma and combining clinical and relevant laboratory examinations (especially tumor markers), the possibility of cavernous hemangioma can be considered while malignant lesions are excluded to eliminate or reduce the risk of surgery and postoperative complications. Our study presents a case of surgical removal of a giant cavernous hemangioma without recurrence at the 2-year follow up.

In summary, pancreatic cavernous hemangioma is a rare tumor with a complex composition that is reflected not only in the imaging examination but also in the immunohistochemical detection, and it can achieve a good outcome by surgical excision.

## Data Availability

The authors declare that all data supporting the findings of this study are available within the article. No datasets were generated or analyzed during the current study.

## Abbreviations

CDFI: Color doppler flow imaging
CEA: Carcinoembryonic antigen
CT: Computed tomography
DSA: Digital subtraction angiography
EUS: Endoscopic ultrasonography
IHC: Immunohistochemical
MRI: Magnetic resonance imaging
